# Mediastinal Lymphadenopathy: Associations Between Computed Tomography Characteristics and Final Etiological Diagnoses in 200 Patients

**DOI:** 10.7759/cureus.109173

**Published:** 2026-05-19

**Authors:** Mohammed Aharmim, Bouchra Badri, Mohamed Lakhal, Nezha Reguig, Jamal Eddine El Bourkadi

**Affiliations:** 1 Department of Pulmonology and Phthisiology, Moulay Youssef Hospital, Rabat, MAR; 2 Department of Pulmonology, Centre Hospitalier Universitaire (CHU) Ibn Sina, Rabat, MAR; 3 Department of Respiratory Diseases, Faculty of Medicine and Pharmacy, Centre Hospitalier Universitaire (CHU) Mohammed VI, Mohammed I University, Oujda, MAR; 4 Department of Pulmonology, Faculty of Medicine and Pharmacy, Moulay Youssef Hospital, Mohammed V University, Rabat, MAR; 5 Department of Pulmonology, Moulay Youssef Hospital, Centre Hospitalier Universitaire (CHU) Ibn Sina, Rabat, MAR

**Keywords:** computed tomography, histopathological diagnosis, lymphoma, mediastinal lymphadenopathy, metastatic lymphadenopathy, reactive lymphadenopathy, sarcoidosis, tuberculosis

## Abstract

Background: Despite recent technological advances in the diagnostic evaluation of mediastinal lymphadenopathy, access to advanced diagnostic procedures remains limited in our setting.

Objective: This study aimed to describe the etiological profile of mediastinal lymphadenopathy and to assess the statistical associations between computed tomography (CT) characteristics of mediastinal lymph nodes and final etiological diagnoses.

Materials and methods: This retrospective observational study was conducted in the Department of Pulmonology at Moulay Youssef University Hospital, Salé, Morocco, over a two-year period. It included all eligible hospitalized patients with mediastinal lymphadenopathy detected on chest CT during the study period. Demographic, clinical, radiological, bronchoscopic, pathological, and follow-up data were collected from medical records. Final diagnoses were established by histopathological confirmation whenever available and, in the remaining cases, by a multidisciplinary assessment integrating clinical, radiological, biological, exposure-related, and follow-up data. Associations between lymph node characteristics and final etiologies were analyzed using the chi-square test, Fisher’s exact test, and the Mann-Whitney test, as appropriate.

Results: A total of 200 patients were included. The mean age was 56 ± 14 years (range: 19-94 years), and women represented 57% of the cohort. Sarcoidosis was the most frequent etiology, observed in 75 patients (37.5%), followed by metastatic lymphadenopathy in 45 (22.5%), reactive lymphadenopathy in 36 (18.0%), tuberculosis in 20 (10.0%), sequelae-related lymphadenopathy in 11 (5.5%), lymphoma in 10 (5.0%), and silicosis in three patients (1.5%). Several CT characteristics were significantly associated with specific etiologies. Sarcoidosis was most frequently associated with bilateral involvement, symmetric distribution, regular margins, homogeneous density, absence of compression, and preferential involvement of stations 3, 4L, 10R, and 10L. Metastatic disease was most frequently associated with unilateral or asymmetric distribution, irregular margins, heterogeneous density, and compression.

Conclusions: This study identified significant associations between selected CT characteristics of mediastinal lymphadenopathy and final etiological diagnoses. These findings may contribute to a more structured radiological assessment and help support the initial etiological orientation in clinical practice. However, they should be interpreted cautiously in light of the retrospective design, the heterogeneous cohort, the absence of systematic histopathological confirmation in all patients, and the lack of formal diagnostic accuracy analyses.

## Introduction

Mediastinal lymphadenopathy is a major diagnostic challenge in daily clinical practice. It may arise from a broad spectrum of conditions, reflecting the wide range of pathological processes that can involve mediastinal lymph nodes. The most common etiologies include inflammatory, infectious, neoplastic, iatrogenic, and autoimmune disorders [1-3].

Because mediastinal lymphadenopathy poses a significant diagnostic challenge in routine practice, and because a substantial radiologic-pathologic correlation is suspected, this study aimed to investigate its etiological profile, with particular focus on the relationship between imaging findings and histopathological results [4-6]. The ultimate goal was to improve diagnostic orientation and optimize management strategies for this complex condition.

Through this work, we aim to contribute to current knowledge in this important area of thoracic medicine and to provide additional insight into a more effective and personalized approach to the management of patients with mediastinal lymphadenopathy [5,6].

## Materials and methods

This retrospective observational study was conducted in the Department of Pulmonology at Moulay Youssef University Hospital, Salé, Morocco, over a two-year period. All eligible hospitalized patients with mediastinal lymphadenopathy detected on chest computed tomography (CT) during the study period were included.

Demographic, clinical, radiological, bronchoscopic, pathological, and follow-up data were collected from medical records using a standardized data collection form. The recorded CT variables included the number of lymph nodes, anatomical location, size, laterality, symmetry, margins, density, calcifications, and compressive effect on adjacent mediastinal structures. Compression was defined as radiological evidence of a mass effect exerted by enlarged lymph nodes on adjacent mediastinal structures on CT imaging.

Bronchoscopy was performed in 126 patients (63.0%). Direct biopsy of a mediastinal lymph node was performed in eight patients (4.0%), while surgical diagnostic procedures, including mediastinoscopy and thoracoscopy, were performed in five patients. In addition, 41.5% of patients underwent biopsy of a peripheral lesion as part of the etiological workup, including peripheral lymph node biopsy in 15 patients (7.5%), bronchial biopsy in 32 (16.0%), CT-guided transthoracic biopsy of a pulmonary lesion in 31 (15.5%), and skin lesion biopsy in 5 (2.5%).

Final diagnoses were established by histopathological confirmation whenever available. In the remaining cases, the final diagnosis was based on a multidisciplinary assessment integrating clinical presentation, radiological findings, biological data, relevant exposure history, and follow-up. This approach was particularly applicable to selected nonmalignant etiologies with characteristic clinicoradiologic features, such as silicosis.

Data were entered into Microsoft Excel 2016 (Microsoft Corporation, Redmond, WA, USA), and statistical analyses were performed using Jamovi software. Quantitative variables were expressed as mean ± standard deviation for normally distributed data and as median with interquartile range for non-normally distributed data. Qualitative variables were expressed as frequency and percentage. Associations between lymph node characteristics and etiologies were analyzed using the chi-square test, Fisher’s exact test, and the Mann-Whitney test, as appropriate. A p-value of less than 0.05 was considered statistically significant. No a priori sample size calculation was performed because this was a retrospective observational study including all eligible patients during the study period.

Ethical considerations

This is a retrospective observational study using anonymized data from routine clinical practice. According to French regulations (Jardé law, Article R1121-1), no IRB approval was required. Patient data were fully anonymized and handled according to CNIL/MRGDR guidelines.

## Results

A total of 200 patients with mediastinal lymphadenopathy detected on chest CT were included in the study. The mean age was 56 ± 14 years (range: 19-94 years), and women represented 57% of the cohort. No relevant past medical history was reported in 59% of patients. Dyspnea was the most frequent presenting symptom (47%), followed by cough (33.5%), while 3% of patients were asymptomatic from a respiratory standpoint. General condition impairment was reported in 39% of cases, and clinical signs of mediastinal compression were present in 7% of patients (Table 1).

**Table 1:** Baseline demographic, clinical, and radiological characteristics of the study population. PET: positron emission tomography; CT: computed tomography.

Variables	Value
Total number of patients	200
Mean age, years	56 ± 14
Age range, years	19-94
Female sex	57%
No relevant past medical history	59%
Dyspnea	47%
Cough	33.5%
Asymptomatic respiratory presentation	3%
General condition impairment	39%
Clinical signs of mediastinal compression	7%
Multiple lymph nodes	97.5%
Bilateral lymphadenopathy	77%
Asymmetric lymphadenopathy	64.5%
Compressive lymphadenopathy	15.5%
Heterogeneous density	41.5%
Calcifications	15.5%
Most frequent location	Station 4R (47.5%)
Median lymph node size, mm	18 (14-25)
Associated pulmonary lesions	83%
PET-CT performed	11 (5.5%)

Regarding CT characteristics, mediastinal lymph nodes were multiple in 97.5% of cases, bilateral in 77%, asymmetric in 64.5%, compressive in 15.5%, heterogeneous in density in 41.5%, and calcified in 15.5%. The most frequent nodal location was the right lower paratracheal station 4R, observed in 47.5% of cases, followed by stations 10R and 10L, present in 44% and 40% of cases, respectively, and by station 7, found in 37.5% of cases. Lymph node size ranged from 10 to 55 mm, with a median short-axis diameter of 18 mm and an interquartile range of 14-25 mm. Associated pulmonary lesions were found in 83% of patients, mainly nodules or micronodules (34%) and consolidations (30%) (Table 1).

Bronchoscopy was performed in 126 patients (63.0%). Direct biopsy of a mediastinal lymph node was performed in eight patients (4.0%), while surgical diagnostic procedures, including mediastinoscopy and thoracoscopy, were performed in five patients (2.5%). In addition, 83 patients (41.5%) underwent biopsy of a peripheral lesion, including peripheral lymph node biopsy in 15 patients (7.5%), bronchial biopsy in 32 (16.0%), CT-guided transthoracic pulmonary biopsy in 31 (15.5%), and skin lesion biopsy in five (2.5%). PET-CT was performed in 11 patients (5.5%) (Table 2).

**Table 2:** Diagnostic procedures performed in the cohort. PET: positron emission tomography; CT: computed tomography.

Diagnostic procedure	n (%)
Bronchoscopy	126 (63.0)
Direct mediastinal lymph node biopsy	8 (4.0)
Mediastinoscopy/thoracoscopy	5 (2.5)
Patients who underwent biopsy of a peripheral lesion	83 (41.5)
Peripheral lymph node biopsy	15 (7.5)
Bronchial biopsy	32 (16.0)
CT-guided transthoracic pulmonary biopsy	31 (15.5)
Skin lesion biopsy	5 (2.5)
PET-CT	11 (5.5)

The etiological distribution of the cohort was as follows: sarcoidosis in 75 patients (37.5%), metastatic lymphadenopathy in 45 (22.5%), reactive lymphadenopathy in 36 (18.0%), tuberculosis in 20 (10.0%), sequelae-related lymphadenopathy in 11 (5.5%), lymphoma in 10 (5.0%), and silicosis in three patients (1.5%) (Table 3). Final diagnoses were established by histopathological confirmation whenever available. In the remaining cases, diagnosis was based on a multidisciplinary assessment integrating clinical presentation, radiological findings, biological data, exposure history when relevant, and follow-up.

**Table 3:** Final etiological diagnoses in the study population.

Etiology	n (%)
Sarcoidosis	75 (37.5)
Metastatic lymphadenopathy	45 (22.5)
Reactive lymphadenopathy	36 (18.0)
Tuberculosis	20 (10.0)
Sequelae-related lymphadenopathy	11 (5.5)
Lymphoma	10 (5.0)
Silicosis	3 (1.5)

In the analytical part of the study, several CT characteristics showed statistically significant associations with final etiological diagnoses. Multiple lymph nodes were most frequently associated with sarcoidosis (χ^2^=18.21, df = 9, Cramer’s V = 0.30, p = 0.033). With regard to nodal location, station 3 (χ^2^=31.37, df = 9, Cramer’s V = 0.40, p < 0.001), station 4L (χ^2^=27.61, df = 9, Cramer’s V = 0.37, p = 0.001), station 10R (χ^2^=40.95, df = 9, Cramer’s V = 0.45, p < 0.001), and station 10L (χ^2^=54.99, df = 9, Cramer’s V = 0.53, p < 0.001) were most frequently associated with sarcoidosis. Lymph node size greater than 20 mm (χ^2^=37.88, df = 9, Cramer’s V = 0.44, p < 0.001) and greater than 10 mm (χ2=51.75, df = 9, Cramer’s V = 0.51, p < 0.001) were also most frequently associated with sarcoidosis.

Unilateral distribution was most frequently associated with metastatic disease (χ^2^=49.20, df = 9, Cramer’s V = 0.50, p < 0.001), whereas bilateral involvement was most frequently associated with sarcoidosis (χ^2^=49.20, df = 9, Cramer’s V = 0.50, p < 0.001). Symmetric distribution was most frequently associated with sarcoidosis (χ^2^=119.66, df = 9, Cramer’s V = 0.78, p < 0.001), while asymmetric distribution was most frequently associated with metastatic disease (χ^2^=119.66, df = 9, Cramer’s V = 0.78, p < 0.001).

Regular margins (χ^2^=99.64, df = 9, Cramer’s V = 0.71, p < 0.001), homogeneous density (χ^2^=84.15, df = 9, Cramer’s V = 0.65, p < 0.001), calcifications (χ^2^=33.98, df = 9, Cramer’s V = 0.41, p < 0.001), and absence of compression (χ^2^=72.67, df = 9, Cramer’s V = 0.60, p < 0.001) were most frequently associated with sarcoidosis. In contrast, irregular margins (χ^2^=108.72, df = 9, Cramer’s V = 0.74, p < 0.001), heterogeneous density (χ^2^=84.15, df = 9, Cramer’s V = 0.65, p < 0.001), and compressive behavior (χ2=72.67, df = 9, Cramer’s V = 0.60, p < 0.001) were most frequently associated with metastatic disease. These statistically significant associations between CT characteristics and final etiological diagnoses are summarized in Table 4.

**Table 4:** Statistically significant associations between CT characteristics and final etiological diagnoses Values are presented as chi-square statistics, degrees of freedom, effect sizes (Cramer’s V), and p-values. A p-value of less than 0.05 was considered statistically significant. CT: computed tomography.

CT characteristics	Category/finding	Most associated etiology	Chi-square	df	Cramer’s V	p-value
Number of lymph nodes	Multiple	Sarcoidosis	18.21	9	0.30	0.033
Laterality	Unilateral	Metastatic disease	49.20	9	0.50	<0.001
Laterality	Bilateral	Sarcoidosis	49.20	9	0.50	<0.001
Symmetry	Symmetric	Sarcoidosis	119.66	9	0.78	<0.001
Symmetry	Asymmetric	Metastatic disease	119.66	9	0.78	<0.001
Margins	Regular	Sarcoidosis	99.64	9	0.71	<0.001
Margins	Irregular	Metastatic disease	108.72	9	0.74	<0.001
Density	Homogeneous	Sarcoidosis	84.15	9	0.65	<0.001
Density	Heterogeneous	Metastatic disease	84.15	9	0.65	<0.001
Calcifications	Present	Sarcoidosis	33.98	9	0.41	<0.001
Compression	Present	Metastatic disease	72.67	9	0.60	<0.001
Compression	Absent	Sarcoidosis	72.67	9	0.60	<0.001
Location	Station 3	Sarcoidosis	31.37	9	0.40	<0.001
Location	Station 4L	Sarcoidosis	27.61	9	0.37	0.001
Location	Station 10R	Sarcoidosis	40.95	9	0.45	<0.001
Location	Station 10L	Sarcoidosis	54.99	9	0.53	<0.001
Size	>20 mm	Sarcoidosis	37.88	9	0.44	<0.001
Size	>10 mm	Sarcoidosis	51.75	9	0.51	<0.001

## Discussion

Mediastinal lymphadenopathy remains a major diagnostic challenge in routine clinical practice because it may reflect a broad range of inflammatory, infectious, neoplastic, iatrogenic, and autoimmune disorders [1-3]. Although lymph nodes measuring at least 10 mm in short-axis diameter have traditionally been considered enlarged, size alone is insufficient for an etiological diagnosis. Chest computed tomography provides additional diagnostic information by assessing nodal distribution, morphology, internal appearance, calcifications, and compressive effect, while positron emission tomography (PET)-CT may further refine diagnostic orientation when available [4-7].

The aim of this study was to investigate the etiological profile of mediastinal lymphadenopathy and to assess the radiologic-pathologic correlation in a resource-limited setting. In our series, the main etiologies were sarcoidosis, metastatic disease, and reactive lymphadenopathy. These findings highlight the wide etiological spectrum of mediastinal lymphadenopathy and support the value of integrating imaging findings with histopathological diagnosis before establishing a final diagnosis [7].

Sarcoidosis was the most common etiology in our cohort. It was significantly associated with bilateral hilar involvement, particularly at stations 10R and 10L, as well as station 4L involvement, nodal size greater than 20 mm, bilateral and symmetrical distribution, regular contours, homogeneous appearance, and absence of compressive effect. These findings are consistent with the typical radiological pattern of sarcoidosis [8,9].

Metastatic lymphadenopathy was the second most frequent etiology and showed significant associations with multiple lymph nodes, unilateral or asymmetrical distribution, heterogeneous internal appearance, irregular contours, compressive effect, and absence of calcifications. These computed tomography features are suggestive of malignant involvement and are consistent with the usual radiological suspicion criteria for metastatic disease [10-12].

Lymphoma was associated with prevascular involvement at station 3A, nodal size greater than 20 mm, compressive effect, heterogeneous appearance, and irregular contours. These results are broadly consistent with the known predilection of lymphoma for anterior mediastinal nodal stations [13-16], with classical staging contributions described in CT-based evaluations [14,15].

Tuberculous mediastinal lymphadenopathy accounted for 10% of cases and was consistently associated with pulmonary parenchymal lesions. It was significantly associated with asymmetrical distribution, heterogeneous appearance, and regular contours, in line with previously reported CT differentiation criteria [17-19], including adult isolated forms [18].

Silicosis was significantly associated with nodal calcifications and preferential involvement of stations 3 and 4L. Calcified mediastinal lymphadenopathy is a recognized feature of pneumoconiosis and may reflect chronic exposure [20-22], including eggshell calcifications [21].

In contrast, reactive lymphadenopathy was characterized by small nodal size, asymmetrical distribution, regular contours, and homogeneous appearance, supporting its consideration in patients with chronic cardiopulmonary, systemic, or interstitial diseases [23-27], particularly in interstitial lung diseases and related conditions [24-26].

PET-CT was performed in only a limited number of patients in our study, which reflects restricted access to this modality in our setting. Consequently, metabolic activity could not be used as a reliable discriminative parameter in our analysis. This limitation underscores the practical value of chest computed tomography-based morphological assessment in environments where advanced imaging and minimally invasive sampling techniques are not consistently available [5,6].

Our findings suggest that several computed tomography features, particularly nodal distribution, size, symmetry, contours, internal appearance, calcifications, and compressive effect, may provide useful preliminary clues to the underlying etiology of mediastinal lymphadenopathy. However, these findings should always be interpreted in conjunction with the clinical context, and histopathological diagnosis remains necessary whenever diagnostic uncertainty persists [1,7].

This study has several limitations. First, it was a single-center retrospective study, which may limit the generalizability of the findings. Second, not all final diagnoses were histopathologically confirmed. In some patients, particularly those with clinicoradiological patterns strongly suggestive of specific nonmalignant etiologies such as silicosis, the final diagnosis was established on the basis of clinical, radiological, biological, exposure-related, and follow-up data. Third, PET-CT was performed in only a limited number of patients, and interobserver agreement for CT interpretation was not assessed. Finally, the reported associations were based on univariate analyses and should not be interpreted as validated diagnostic performance measures [28].

## Conclusions

This retrospective study identified significant associations between selected CT characteristics of mediastinal lymphadenopathy and final etiological diagnoses. These findings may help provide a more structured radiological assessment and contribute to initial etiological orientation in clinical practice. However, the results should be interpreted cautiously given the retrospective design, the heterogeneous cohort, the absence of systematic histopathological confirmation in all cases, and the lack of formal diagnostic accuracy analyses. Larger multicenter studies are needed to further validate these associations.
